# Chloroform Fraction of *Centratherum anthelminticum* (L.) Seed Inhibits Tumor Necrosis Factor Alpha and Exhibits Pleotropic Bioactivities: Inhibitory Role in Human Tumor Cells

**DOI:** 10.1155/2012/627256

**Published:** 2012-02-28

**Authors:** Aditya Arya, Mouna Achoui, Shiau-Chuen Cheah, Siddig Ibrahim Abdelwahab, Putri Narrima, Syam Mohan, Mohd Rais Mustafa, Mustafa Ali Mohd

**Affiliations:** ^1^Department of Pharmacology, Faculty of Medicine, University of Malaya, 50603 Kuala Lumpur, Malaysia; ^2^Department of Pharmacy, Faculty of Medicine, University of Malaya, 50603 Kuala Lumpur, Malaysia

## Abstract

We investigated the antioxidant potential, cytotoxic effect, and TNF-*α* inhibition activity with NF-*κ*B activation response in a chloroform fraction of *Centratherum anthelminticum* seeds (CACF). The antioxidant property of CACF was evaluated with DPPH, ORAC, and FRAP assays, which demonstrated significant antioxidant activity. The cytotoxicity of CACF was tested using the MTT assay; CACF effective inhibitory concentrations (IC_50_) for A549, PC-3, MCF-7, and WRL-68 cells were 31.42 ± 5.4, 22.61 ± 1.7, 8.1 ± 0.9, and 54.93 ± 8.3 *μ*g/mL, respectively. CACF effectively and dose-dependently inhibited TNF-*α* release, *in vitro* and *in vivo*. CACF inhibited TNF-*α* secretion in stimulated RAW264.7 macrophage supernatants with an IC_50_ of 0.012 *μ*g/mL, without affecting their viability; the highest dose tested reduced serum TNF-*α* by 61%. Acute toxicity testing in rats revealed that CACF was non-toxic at all doses tested. Matching the cytotoxic activity towards a mechanistic approach, CACF dose-dependently exhibited *in vitro* inhibitory effects against the activation of NF-*κ*B translocation in MCF-7 cells. Preliminary phytochemical screening with GC/MS analysis detected 22 compounds in CACF, of which morpholinoethyl isothiocyanate was the most abundant (29.04%). The study reveals the potential of CACF in the treatment of breast cancer and in oxidative stress conditions with associated inflammatory responses.

## 1. Introduction

Since time immemorial, traditional herbs have been used as remedies for several diseases [1]. Even though new synthetic drugs are available, traditional medicine is still being utilized today as part of primary healthcare in several parts of the world. Traditional medicine is often sought because it is economical, readily available, and trusted by its advocate [2]. Studies on herbal medicines have shown the positive correlation of traditional claims and scientific data [3]. Some of these traditional treatments are part of clinical practice, and few are found to be not beneficial [4]. Support for traditional medicine and the numerous natural products with biological activity have led to multidisciplinary investigations utilizing preliminary screening procedures as well as advanced mechanistic studies to develop drugs which may be used clinically.

*Centratherum anthelminticum* (L.) KUNTZE (family: Asteraceae) is an erect, pubescent annual herb found widely in the Indian subcontinent which is locally known as “Somraj,” and its seeds are known as “Kalijiri” in Hindi [5, 6]; scientific synonyms for this plant include *Vernonia anthelmintica* and* Conyza anthelmintica*, among others. This plant is used extensively in Ayurveda for the treatment of cough and diarrhoea, as well as an anthelmintic, stomachic, diuretic, and antiphlegmatic agent [5, 7]. Experimental studies proved the pharmacological effects of this plant's seeds extract, including antihelminthic [8], larvicidal [9], antipyretic [10], antifilarial [11], antihyperglycemic [12], antimicrobial [13], and diuretic [14] activities. In addition to primary metabolites, the seeds of this plant contain glycosides, phenolic compounds, tannins, flavonoids, saponins, and sterols [15]. Examples of secondary metabolites found in *C. anthelminticum* include the following: flavonoids such as 2′,3,4,4-tetrahydroxychalcone (Butein); 5,6,7,4′-tetrahydroxy flavone and 7,3′,4′-trihydroxydihydroflavone [16]; sterols such as sterol-4-alpha-methylvernosterol, vernosterol, and avernosterol [17]; steroids such as (24a/R)-stigmasta-7-en-3-one, 24(a/R)-stigmasta-7, 9(11)-dien-3-one, 24(a/S)-stigmasta-5 and 22-dien-3*β*-ol, stigmasta-7, and 22-dien-3*β*-ol [18].

While several studies investigated the pharmacological effects of this plant, the antiinflammatory and anti-cancer activities are yet to be investigated. Inflammation is a natural defense mechanism by the host which plays a role in various diseases. Immune cells are particularly important in inflammation because they orchestrate the release of several mediators such as cytokines, prostaglandins, and nitric oxide, which play a part in the defense process. However, uncontrolled production of these mediators is associated with tissue damage due to oxidative stress [19]. Oxidative stress resulting from reactive oxygen and nitrogen species (ROS and RNS) was shown to cause DNA mutations and cell death and affect cell proliferation. Cells which survive the DNA damage caused by oxidative stress are likely to have aberrant repair mechanisms, the proliferation of these genetically instable cells might eventually progress toward carcinogenesis. Therefore, this study looked into the antioxidant approaches, the *in vitro* and *in vivo* TNF inhibition activity counting cytotoxic effect in the chloroform fraction with further evaluation of linkage between NF-*κ*B activation on breast cancer (MCF-7) cell lines.

## 2. Materials and Methods

### 2.1. Cell Lines and Reagents

All cell lines were purchased from ATCC (Rockville, MD, USA). RPMI medium, penicillin and streptomycin solution, and phosphate buffer Saline (PBS) were purchased from Invitrogen (Rockville, MD, USA). MTT, DMSO, and heat-inactivated fetal bovine serum and 0.25% trypsin solution were purchased from Sigma-Aldrich Chemicals (Saint Louis, MO, USA). Cell culture treated 96-well plates, and cell culture flasks were purchased from Orange Scientific (Braine-l'Alleud, Belgium). PBS 75 nM, pH Dulbecco's Modified Eagle Medium (DMEM), phosphate buffered saline, Hanks' balanced salt solution (HBSS), and 3-(4,5-Dimethylthiazol-2-yl)-2,5-diphenyltetrazolium bromide (MTT) were from Invitrogen (Carlsbad, USA). Fetal bovine serum (FBS), LPS from *E. coli* serotype 0111:B4, and dimethylsulfoxide (DMSO) were obtained from Sigma (St. Louis, USA). Murine TNF-*α* ELISA kit was from eBioscience (San Diego, USA). NF-*κ*B activation kit from Thermo Scientific Cellomics, fluorescence sodium salt, DPPH and FRAP reagents, Pentoxifylline and Paclitaxel (Sigma-Aldrich), AAPH, Quercetin, and Trolox were from Sigma-Aldrich Chemicals (S. Louis, MO, USA). Plates were read using Chameleon V Multilabel microplate reader (Hidex, Turku; Finland) in 96-well format black plate.

### 2.2. Plant Materials

The seeds of the plant *Centratherum anthelminticum *were procured from the medicinal plant cultivation zone of Amritum Bio-Botanica Herbs Research Laboratory Pvt. Ltd, Betul, Madhya Pradesh, India. The seeds of the plant were authenticated by the Quality Control department of the company itself. Voucher specimen (CA-9) is deposited in the Pharmacology Department of the University of Malaya, Malaysia.

### 2.3. Extraction Procedure

**Figure 1:**
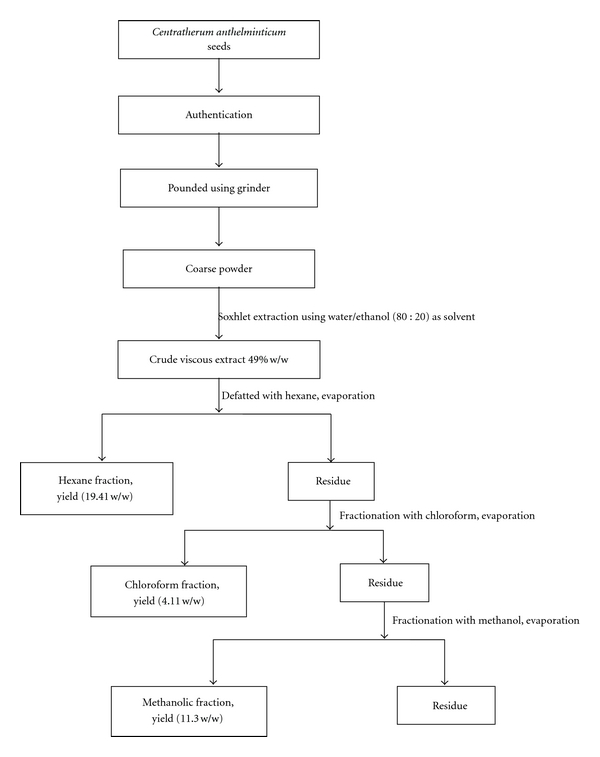
Schematic diagram of extraction/fractionation of *C. anthelminticum* seeds.

The coarsely powdered seeds (100 g) were extracted with water : ethanol (80 : 20) using a Soxhlet extractor for 24 h (Figure 1). The solvent was completely evaporated using rotary evaporator. The brown viscous crude extract weighing (49% w/w) obtained was further fractionated successively with hexane, chloroform and methanol, then the solvent from each fraction was completely recovered with the help of rotary evaporator under reduced pressure. After drying, the final yields with hexane (CAHF), chloroform (CACF), and methanol fraction (CAMF) were (19.41% w/w), (4.11 w/w), and (11.3% w/w), respectively. Then the dried fractions were kept below −20°C before being used. The in pilot bioactivity testing, chloroform fraction (CACF) had shown the positive effect and hence was chosen for further analysis.

### 2.4. Animals

Altogether 72 Sprague Dawley rats of 150–200 g were obtained from the University of Malaya Medical Centre Animal House and maintained under standard conditions of lighting (12 h of light and darkness) and nutrition (food and water *ad libitum*) throughout the experimental period. Studies were performed in accordance with the Medical Research Council Guidelines on Ethics in Animal Experimentation. This study was approved by the Animal Experimentation ethics committee at the University of Malaya Medical Center-(UMMC), Animal Ethics no: FAR/10/11/2008/AA(R).

### 2.5. Total Phenolic Content (TPC)

TPC of CACF was determined using Folin-Ciocalteu method [20]. CACF was prepared in a concentration of 10 mg/mL in methanol. Five microliters of this solution were transferred to 96-well mircoplate (TPP, USA). To this, 80 *μ*L of Folin-Ciocalteu reagent (1 : 10) was added and mixed thoroughly. After 5 min, 160 *μ*L of sodium bicarbonate solution (NaHCO_3_ 7.5%) was added, and the mixture was allowed to stand for 30 min with intermittent shaking. Absorbance was measured at 765 nm using microplate reader (Molecular Devices, Sunnyvale, USA). The TPC was expressed as gallic acid equivalent (GAE) in mg/g extract and obtained from the standard curve of gallic acid. The gallic acid standard curve was established by plotting concentration (mg/mL) versus absorbance (nm) (*y* = 0.001*x* + 0.045; *R* ^2^ = 0.9975), where *y* is absorbance and *x* is concentration in GAE (*n* = 3).

### 2.6. Antioxidant Activity of C. anthelminticum

#### 2.6.1. 1,1-Diphenyl-2-picrylhydrazyl (DPPH) Radical Scavenging Activity of CACF

The scavenging activity of CACF on DPPH was determined using the method described in [21]. This method is based on the reduction of purple DPPH to a yellow-colored diphenyl picrylhydrazine. Changes in color were measured at 518 nm. CACF was tested at final concentrations ranging from 600 to 10 *μ*g/mL in ethanol. One milliliter of 0.3 mM DPPH ethanol solution was added to 2.5 mL of sample solution of different concentrations to make the test solutions, while 1 mL of ethanol was added to 2.5 mL of samples to make the blank solutions. The negative control (blank) consisted of 1 mL DPPH solution plus 2.5 mL of ethanol. These solutions were allowed to react at room temperature for 30 minutes in the dark. The absorbance values were measured at 518 nm and converted into percentage antioxidant activity using the following equation:

(1) $$\text{\%}\;\text{Inhibition}=\left[\frac{\left(A_{B}-A_{A}\right)}{A_{B}}\right]\times 100,$$

where *A*_*B*_ is the absorption of blank sample; *A*_*A*_ is the absorption of tested samples.

The IC_50_ as well as the kinetics of DPPH scavenging activity was determined. Ascorbic acid and butylated hydroxytoluene (BHT) was used as a positive control in this assay.

#### 2.6.2. ORAC Antioxidant Activity Assay

The oxygen radical absorbance capacity (ORAC) assay was done based on the procedure described earlier by Choi et al. [21] with slight modifications. Briefly, 175 *μ*L of the sample/blank were dissolved with PBS at concentrations of 160 *μ*g/mL, pH 7.4. Serial dilutions of the standard Trolox were prepared from 75 mM. The assay was performed in 96-well black microplates 25 *μ*L of samples (CACF), standard (Trolox), blank (solvent/PBS), or the positive control (quercetin) was added to the wells. Subsequently, 150 *μ*L of fluorescent sodium salt solution was added, and the plate was then incubated for 45 minutes at 37°C. Twenty five microliters of 2,20-azobis (2-amidinopropane) dihydrochloride (AAPH) solution was added for a total volume of 200 *μ*L/well. Fluorescence was recorded until it reached zero (excitation at 485 nm, emission at 535 nm) using a fluorescence spectrophotometer (Perkin—Elmer LS 55), equipped with an automatic thermostatic autocell holder at 37°C. Data were collected every 2 mins for a duration of 2 hrs and were analyzed by calculating the differences of areas under the fluorescein decay curve (AUC) between the blank and the sample. Values were expressed as Trolox equivalents.

#### 2.6.3. Ferric Reducing/Antioxidant Power (FRAP) Assay

The FRAP assay was slightly modified from the method of Benzie and Strain [22]. The stock solutions included 300 mM acetate buffer (pH 3.6), 10 mM TPTZ (2,4,6-tripyridyl-s-triazine) solution in 40 mM HCl, and 20 mM FeCl_3_ *·*6H_2_O. The fresh working solution was prepared by mixing 25 mL acetate buffer, 2.5 mL TPTZ, and 2.5 mL FeCl_3_ *·*6H2O. The temperature of the solution was raised to 37°C before use. CACF (10 *μ*L) was allowed to react with 190 *μ*L of the FRAP solution for 30 min in the dark. Colorimetric readings of the product ferrous tripyridyltriazine complex were taken at 593 nm. The standard curve was linear between 200 and 1000 *μ*M FeSO_4_. Results are expressed as *μ*M Fe (II)/g dry mass and compared with those of Ascorbic acid and BHT.

### 2.7. Cell Viability and Cytotoxicity

#### 2.7.1. Cell Culture

All cell lines were obtained from American Type Cell Collection (ATCC) and maintained in a 37°C incubator with 5% CO_2_ saturation. Human breast carcinoma (MCF-7) and normal hepatic (WRL-68) cell lines were maintained in Dulbecco's modified Eagle's medium (DMEM), while nonsmall cell lung cancer cells (A549) and prostate adenocarcinoma cells (PC-3) were maintained in RPMI medium. Both media were supplemented with 10% fetus calf serum (FCS), 100 units/mL penicillin, and 0.1 mg/mL streptomycin. Cells were cultured using standard aseptic techniques and were seeded at the indicated densities below.

#### 2.7.2. Cellular Viability

The inhibitory effects of CACF on the indicated cell lines' growth were tested using the MTT assay [23]. Cells were seeded at a density of 1 × 10^5^cells/mL in a 96-well plate and incubated for 24 hours at 37°C and 5% CO_2_. Cells were then treated with CACF and incubated for another 24 hours, after which the MTT solution at 2 mg/mL was added for 1 hour. The insoluble formazan product was dissolved in DMSO, and absorbance was measured at 570 nm using Plate Chameleon V microplate reader (Hidex, Turku, Finland). Results were expressed as a percentage calculated from the ratio of absorbance of treated cells to untreated cells. The concentration that caused a 50% loss of cell growth (IC_50_) was used to measure the CACF growth inhibition potency.

### 2.8. TNF-*α* Inhibition Activity of CACF

#### 2.8.1. *In Vitro* Cell Viability and TNF-*α* Production Assays

 Murine macrophage cells RAW 264.7 were seeded in 96-well plates at 5 × 10^5^cells/mL. Cells were either left untreated in DMEM or pretreated with CACF at the indicated concentrations for 30 minutes. Cell stimulation, viability, and TNF production measurements were conducted exactly as in [24]. Percentage viability was calculated as follows: cell viability (%) = [(OD_570_ (sample)/OD_570_ (control)) ×100]. 

Whereas percentage TNF inhibition was calculated as follows:

(2) $$\text{\%}\;\text{inhibition}=100\times \left[\frac{\left(\left[\text{TNF}\right]\;\text{control}-\left[\text{TNF}\right]\;\text{sample}\right)}{\left[\text{TNF}\right]\;\text{control}}\right],$$

where control indicates cells treated in LPS alone.

#### 2.8.2. Measurement of *In Vivo* Serum TNF

Healthy male rats were selected and divided into 6 groups (*n* = 8 for each group). The rats were pretreated with the indicated solutions for 30 minutes before lipopolysaccharide (LPS) stimulation: group 1 was pretreated intraperitoneally (i. p.) with 1 mL of PBS alone, group 2 with dexamethasone (6 mg/kg BW) in 25% DMSO, group 3 with CACF (25 mg/kg BW) in 25% DMSO, group 4 with CACF (50 mg/kg BW) in 25% DMSO, and group 5 with CACF (100 mg/kg BW) in 25% DMSO. Groups 1–5 were treatment groups stimulated with LPS, while group 6 made up the untreated negative control in which rats were given a solution of 25% DMSO in PBS and no LPS. Subsequent to pretreatment, LPS (1 mg/kg) was then administered in 1 mL of pyrogen-free normal saline i. p for five treatment groups, and PBS was administered i. p. for the negative control group. Blood was withdrawn from the animals under ether anesthesia after 90 minutes of LPS or PBS administration. Serum was collected and stored at −80°C until analysis. Serum levels of TNF-*α* were determined using rat TNF-*α* ELISA kit according to the manufacturer's protocol (e Bioscience, San Diego, USA).

### 2.9. Acute Toxicity Study

Healthy adult rats of either sex were divided into 4 groups (*n* = 6) and were orally fed with increasing doses of CACF: 10, 20, 100, and 1000 mg/kg body weight (BW). The rats were observed continuously for 2 hours for behavioral, neurological, and autonomic profiles and after 24 and 72 hours for any lethality. All procedures were according to the guidelines stated by OECD.

### 2.10. NF-*κ*B Translocation Assay

NF-kB translocation in MCF-7 cells was examined using NF-kB activation HCS kit which contains Hoechst 33342 and Alexa Fluor 488 conjugated anti-NF-kB dyes. MCF-7 cells were seeded into 96-well plates (Perkin-Elmer Inc., Wellesley, MA, USA) at 6000 cells/well. After overnight, cells were treated with different concentrations of CACF for 1 hr, followed by treatment with 10 *μ*g/mL TNF-*α* for another 30 minutes. Fixation, permeabilization, and immunofluorescence staining of cells were performed according to the manufacturer's instructions. ArrayScan reader was used to quantify the difference between the intensity of nuclear and cytoplasmic NF-*κ*B-associated fluorescence, reported as translocation parameter.

### 2.11. Identification and Chemical Analysis Using GC-MS

Gas chromatography mass spectrometry (GC-MS) analysis of the selected fraction was carried out on a Shimadzu GC-17 A network GC system coupled to a mass-selective detector: MS-QP50-50. Separation was conducted on an HP-5 MS column (30 m × 0.32 mm × 3.0 *μ*m), with helium as the carrier gas at a flow rate of 1.0 mL/min. The injection volume was 1 *μ*L with a split ratio of 10 : 1. The column temperature was initially held at 100°C for 3 min and then increased to 290°C at a rate of 10°C/min. The column temperature was then maintained at 290°C for 3 min. The temperatures of the injector and detector were 250°C and 280°C, respectively. Mass acquisition was performed in the range of 40–550 atomic mass units (a. m. u) using electron impact ionization at 70 eV. The major components in this sample were predicted by a spectral database matching against the library of National Institute of Standards and Technology (NIST21 and NIST Wiley).

### 2.12. Statistical Analysis

Experimental values were expressed as the means ± standard deviation (SD) of the number of experiments indicated in the legends. Statistical significance was assessed using one-way analysis of variance (ANOVA) followed by a multiple comparison test (Tukey's post-hoc test), were *P* < 0.001, *P* < 0.01, and *P* < 0.05 were considered significant. Pearson correlation coefficient was used to assess the correlation between phenolic content and antioxidant activities.

## 3. Results

### 3.1. Antioxidant Activity

Table 1 provides a summary of the antioxidant and total phenolic content of CACF. The total phenolic content of CACF was determined to be 37.16 ± 0.85 *μ*g GAE/mg extract.

#### 3.1.1. DPPH Scavenging Activity of CACF

CACF exhibited a significant dose-dependent inhibition of DPPH activity (*P* < 0.05), with an IC_50_ value of 22.56 ± 1.4 *μ*g/mL (Table 1). Maximal DPPH scavenging activity occurred at 41 ± 1.2 *μ*g/mL of CACF with an inhibition of 89%.

#### 3.1.2. Ferric Reducing Antioxidant Power of CACF

CACF showed a significant dose-dependent FRAP value (*P* < 0.05) with a 1048.3 *μ*mol/L for the fraction, while the positive control used in this study displayed a value of 6240 and 907.7 *μ*mol/L for ascorbic acid and BHT, respectively (Table 1).

#### 3.1.3. ORAC Activity of CACF

The area under the curve (AUC) was calculated for oxygen radical absorbance capacity of CACF, trolox, and the positive control quercetin. ORAC results are demonstrated in Table 1. CACF had an ORAC value of 992.34 ± 45.12 *μ*M trolox equivalent at 20 *μ*g/mL. On the other hand, quercetin had an ORAC value of 1018.00 ± 34.82 *μ*M of Trolox equivalent at 5 *μ*g/mL.

### 3.2. Cytotoxic Activity of CACF

**Figure 2:**
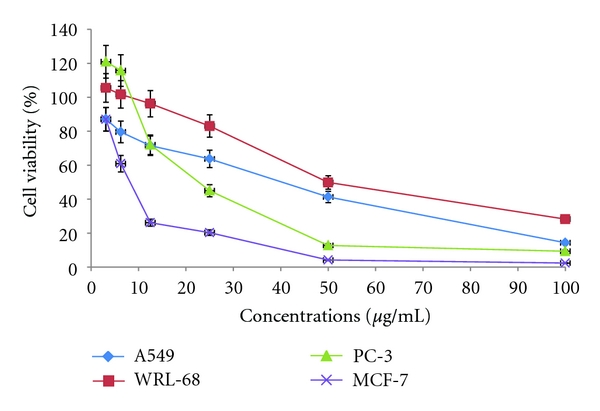
CACF was tested with a series of different doses on A549, MCF-7, PC-3, and WRL-68 cells, respectively. After 24 hours, cell viability was determined by the MTT assay. Test agents induced cell cytotoxicity in a concentration-dependent manner. These dose titration curves allowed determining IC_50_ for the test agents towards different cell lines. The IC_50_ value of CACF on the viability of A549, PC-3, MCF-7, and WRL-68 has been determined to be 31.42 ± 5.4, 22.61 ± 1.7, 8.1 ± 0.9, and 54.93 ± 8.3 *μ*g/mL, respectively.

To evaluate the cytotoxic activity, CACF was tested with a series of different doses on nonsmall cell lung cancer (A549), prostate cancer (PC-3), breast cancer (MCF-7), and normal hepatic cells (WRL-68), respectively. After 24 hours, cell viability was determined by the MTT assay. CACF induced cell cytotoxicity in a concentration-dependent manner. These dose titration curves allowed determination of IC_50_ for the CACF towards different cell lines. CACF demonstrated dose-dependent cytotoxic effects with IC_50_ values of 31.42 ± 5.4, 22.61 ± 1.7, 8.1 ± 0.9, and 54.93 ± 8.3 *μ*g/mL; in A549, PC-3, MCF-7 and WRL-68, respectively (Figure 2). In line with these screening, reference drug (Paclitaxel) was used as a positive control whose IC_50_ on tested A549, PC-3, MCF-7 and WRL-68 were 5.675 ± 1.03, 0.37 ± 0.03, 1.583 ± 0.24, and 0.666 ± 0.05, respectively. Hence, these results point out that cell lines vary in their sensitivity.

### 3.3. *In Vitro* Inhibitory Effects of CACF on TNF Production and RAW264.7 Cell Viability

**Figure 3:**
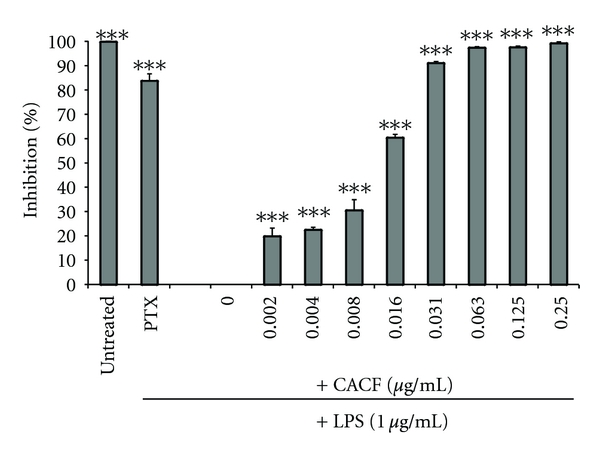
The effects of CACF on TNF production in RAW264.7 cells. Cells were pretreated with the indicated concentrations of CACF, or the TNF inhibitor pentoxifylline (PTX). The cells were stimulated with LPS (1 *μ*g/mL) for four hours or were left untreated (DMSO). The protein concentration was measured using ELISA. Data is representative of three independent experiments and was analyzed using one-way ANOVA with Tukey's post hoc test. The inhibitory effect of CA chloroform fraction was significantly different from stimulated cells (LPS) (***P < 0.001).

**Figure 4:**
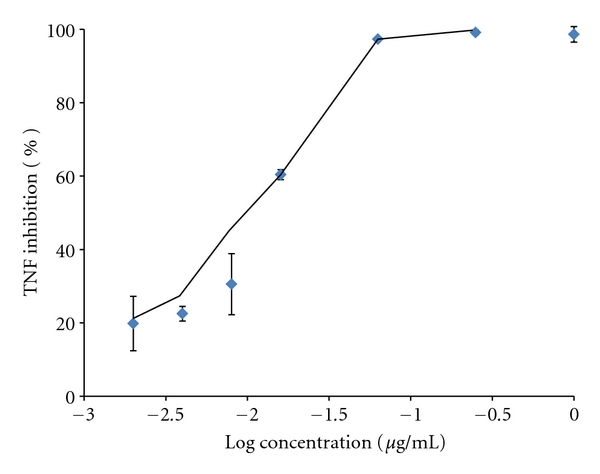
Dose-response effect of the CACF on TNF inhibition: the chloroform fraction had an IC_50_ of 0.012 *μ*g/mL (LOG IC_50_: −1.931).

**Figure 5:**
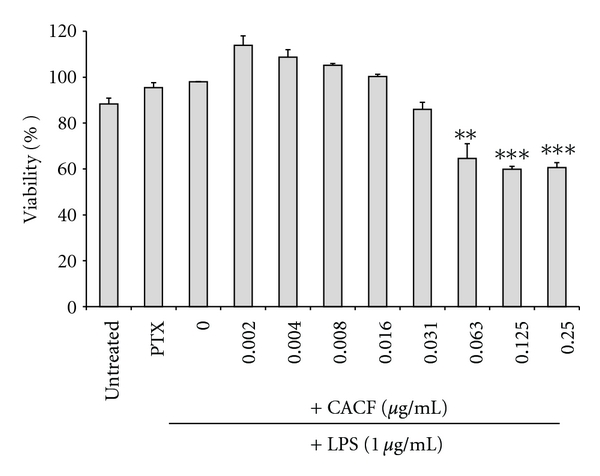
The effects of CACF on RAW264.7 cells' viability. Cells were pretreated with the indicated concentrations of CACF for 4 hours or were left untreated (DMEM). Data is the average of three independent experiments (± SD) and was analyzed using one-way ANOVA with Tukey's posttest (*P < 0.05, **P < 0.01, and ***P < 0.001).

Stimulation of RAW264.7 with LPS for 4 hours caused a significant increase in TNF-*α* production (Figure 3). CACF effectively and dose dependently inhibited TNF-*α* release with an IC_50_ of 0.012 *μ*g/mL as depicted in Figure 4. CACF exhibited maximal TNF inhibition of 90% at 0.31 *μ*g/mL. This significant inhibitory effect was observed at noncytotoxic doses ranging from 0.031 to 0.002 *μ*g/mL (Figure 5).

### 3.4. *In Vivo* Activity

#### 3.4.1. Acute Toxic Effects of CACF

The acute toxicity study revealed the nontoxic nature of CACF. There was a 100% survival rate of the animals treated with CACF at doses ranging from 10 to 1000 mg/kg. Moreover, no toxic effects were observed throughout the study.

#### 3.4.2. The Inhibitory Effect of CACF on Serum TNF

**Figure 6:**
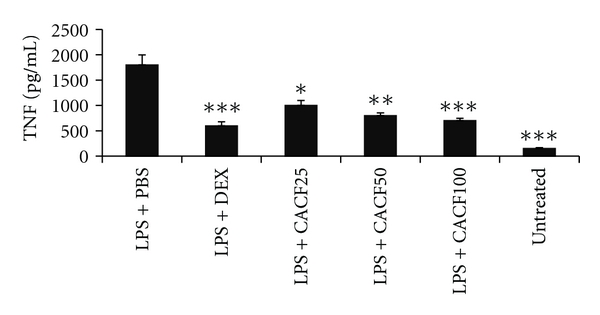
Effect of CACF on serum TNF levels in rats. Different treatment groups were pretreated i.p with 25, 50, and 100 mg/kg of (LPS + CACF), 6 mg/kg of dexamethasone (LPS + DEX) or with phosphate buffer saline (LPS + PBS), and DMSO (Untreated) for 30 mins. All the treatment groups were then either injected with 1 mg/kg of lipopolysaccharides (LPS) or with PBS for 90 mins. Blood was withdrawn, and serum TNF was quantified using ELISA. One-way ANOVA with Tukey's post-analysis was used to calculate the statistical significance among the groups when compared to LPS + PBS. ***P < 0.001, **P < 0.01, and *P < 0.05.

Stimulation of animals with LPS leads to an increase in serum TNF levels up to 2 ng/mL in rats pretreated with PBS alone. On the other hand, animals pretreated with dexamethasone and CACF have shown a significant reduction in TNF levels (Figure 6). Moreover, CACF showed a dose-dependent inhibitory effect of serum TNF in LPS-stimulated rats, and this effect in rats pretreated with 100 mg/kg was almost 61%, to that of dexamethasone 67%, respectively.

### 3.5. CACF Inhibits NF-kB Activity

**Figure 7:**
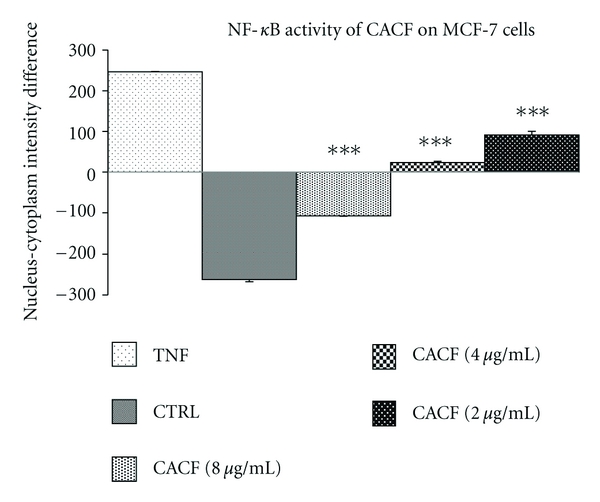
Dose-response histogram of CACF treated MCF-7 cells for 1 hours and then stimulated for 30 minutes with 10 ng/mL TNF-*α* for quantitative image analysis of intracellular targets.

**Figure 8:**
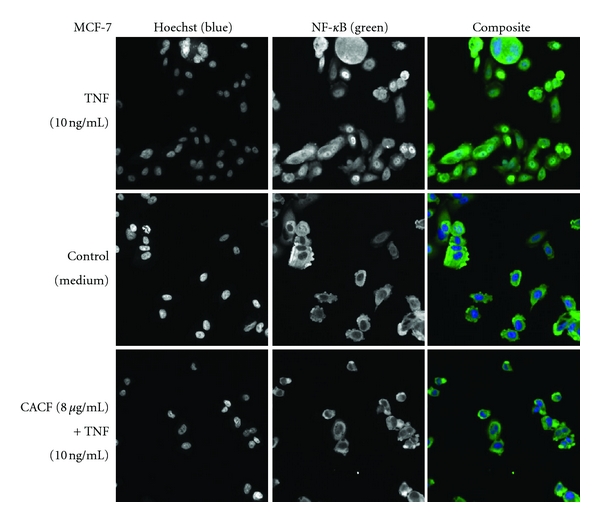
Stained MCF-7 cells were treated with CACF (8 *μ*g/mL) for 1 hour and then stimulated for 30 minutes with 10 ng/mL TNF-*α* (NF-*κ*B activation).

In this study, we tested CACF for its *in vitro *inhibitory effects against NF-*κ*B translocation activated by TNF-*α* and illustrated by HCS assay. CACF displayed significant inhibitory effects on the activation of NF-*κ*B (Figure 7). In parallel, the morphological changes of NF-*κ*B translocation indicated by immunofluorescence staining (Figure 8) showed an inhibitory effect of CACF on TNF-*α*-induced NF-*κ*B translocation in a dose-dependent manner. When cells remain untreated, most of the fluorescence staining for NF-*κ*B were in the cytoplasm and rare NF-*κ*B staining found in nuclei area. While cells were stimulated with the TNF-*α* alone, NF-*κ*B staining significantly increased in nuclei area, suggesting that NF-*κ*B translocated from cytoplasm into the nucleus. However, MCF-7 cells were treated with 8, 4, and 2 *μ*g/mL of CACF, and NF-*κ*B translocation induced by TNF-*α* was inhibited.

### 3.6. Chemical Composition

The possible chemical composition of CACF analyzed by GC/MS is presented in (Table 2). Based on the similarity index out of major peaks, total 22 compounds were detected in this fraction. The most abundant component comprise of 2-Morpholinoethyl isothiocyanate (29.04%), 1,E-11,Z-13-Octadecatriene (16.48%), and Octadecanoic acid, butyl ester (16.15%). Eight compounds (<5%) were not reported in Table 2 due to their minority.

## 4. Discussion

The current study revealed the pleiotropic bioactivities of the chloroform fraction of *Centratherum anthelminticum *(CACF) seeds. The antioxidant assays performed using the DPPH, ORAC, and FRAP methodologies revealed the free radical-scavenging possibilities of this fraction. Antioxidants are substances that may protect cells from the damage caused by unstable molecules known as free radicals. Several factors can lead to the accumulation of free radicals in the cell; examples are certain chemicals, ultraviolet radiations, inflammatory cytokines, and bacterial lipopolysacharides, and other pesticides further generate oxidative stress conditions and extend their contribution in the progression of various ailments including neurodegenerative diseases, atherosclerosis, diabetes, inflammation, and carcinogenesis [19, 25–27]. However, present work demonstrated that CACF fraction is grouped with certain antioxidant compounds. Our results warrant the probability of the CACF as a natural source of antioxidants which could be promising in hunting free radicals and treating diseases related to free radical reactions. Hence, the ability to scavenge free radicals may attenuate the several signalling pathways triggering tissue damage and inflammation, which in turn will have a protective effect on the cells [28, 29].

Having uncovered the antioxidant nature of CACF, we then turned to look at its effects in a living cell system. Cytotoxic screening models provide important preliminary data to select plant extracts or natural compounds with potential anticancer properties [30]. In this study, the cytotoxic effect of CACF was investigated by the addition of the MTT tetrazolium salt [23] to various cancer cell lines previously treated with CACF. CACF showed selective cytotoxic effect on MCF-7 compared with other tested cell lines with low IC_50_ value, nevertheless it was not cytotoxic to normal cell line, WRL-68. These results specify that cell lines differ in their sensitivity to the same CACF, which may be determined by multiple-cell type-specific signalling cascades and transcription factor activities [31].

In addition to that, CACF exhibited inhibition of TNF production both *in vitro* and *in vivo* without affecting cell viability and animal survival, respectively. This also warrants the ethnomedical uses of this plant in the treatment of inflammatory conditions. The ability of CACF to inhibit TNF production may attribute to several factors. Previous studies on *C. anthelminticum *have shown the presence of several bioactive components [18], for example, butein which is one of the secondary metabolites isolated from the seeds of this plant shown to possess both antioxidant and anti-inflammatory activities [14]. The ability of CACF to interfere with inflammatory signalling may in turn explain its cytotoxic effects on cancer cells, since these pathways are also involved in the survival, proliferation, invasion, angiogenesis and metastasis of tumours [32].

Moreover, this fraction along with pleotropic bioactivities demonstrated the *in vitro* inhibitory effects on cell proliferation in MCF-7 cells, which moves our line of study towards carrying out additional experiments to get a better understanding in elucidating mechanisms of breast carcinogenesis with possible future strategies. It is well known that antioxidants are known for their ability to scavenge free radicals from the various stimuli, resulting in activation of transcription factors involved in the transcription of survival and inflammatory genes such as nuclear factor kappa B (NF-*κ*B) [24]. NF-*κ*B is a transcription factor involved in copious inflammatory and cancer-related ailments and has developed as a foremost target in drug discovery [33]. The consequences of NF-*κ*B transcription factors in constitutive activation includes increased survival signalling, cell proliferation, angiogenesis, and invasion, which are key features of the malignant phenotype [34]. Our study demonstrated that CACF in a concentration-dependent manner inhibited constitutive activation of NF-kB translocation stimulated by TNF-*α* in MCF-7 cells. TNF-*α*-induced transcript levels for the adhesion molecules, and this might interfer at an early stage of signaling event induced by TNF-*α*. This suggests that CACF may inhibit the expression of the cell adhesion molecules by interfering with the transcription of their respective genes and may inhibit either the initiation of transcription or the stability of the mRNAs encoding in these molecules. It is well understood that NF-*κ*B is an important transcription factor involved in the gene regulation and contributing in immune and inflammatory responses, including genes encoding [35, 36]. Moreover, tumors with constitutive NF-kB activity have inherent resistance to many anticancer therapies. Thus, NF-*κ*B is believed to play an important role in the regulation of inflammatory response associated with cancer therapy [37, 38].

Analysis of the nonpolar extractable of plant material using GC/MS has been applied before [39]. Our results demonstrate that CACF contains various bioactive components such as 2-morpholinoethyl isothiocyanate which represents 29.04%. Isothiocyanates have been shown to inhibit carcinogenesis and also useful as chemopreventive agents against cancers. They work on a variety of levels. These compounds are shown to induce apoptosis in certain cancer cell lines and in some cases and are even able to induce apoptosis in cells that are resistant to some currently used chemotherapeutic drugs [40–44].

In summary, the chloroform fraction of *Centratherum anthelminticum* seed (CACF) displayed a range of interrelated *in vitro* activities, ranging between antioxidant, cytotoxic and *in vitro* and *in vivo* TNF-*α* inhibition while remaining safe. Furthermore, CACF is maintained concerning mechanistic approach linked with cytotoxic activity on targeted cell line which positively inhibited NF-kB translocation in MCF-7 cells. Therefore, these results merit further pharmacological investigations with detailed phytochemical analysis on *C. anthelminticum*.

## Figures and Tables

**Table 1:** Antioxidant activity profile of the chloroform fraction of *Centratherum anthelminticum *(CACF).

Samples	DPPH IC_50_, *μ*g/mL	FRAP (*μ*mol/L)	ORAC Equivalent conc. Trolox (20 *μ*g/mL (*μ*M)** IC_50_		TPC *μ*g GAE/mg
CACF	22.56^a^ ± 1.4	1048.3^a^ ± 21.2	992.34^a^ ± 45.12	86.62	37.16 ± 0.85
Ascorbic acid	15^a^ ± 0.3	6240^b^ ± 56.2	—	—	—
BHT	17^a^ ± 0.4	907.7^a^ ± 54.8	—	—	—
Quercetin	—	—	1018.00^b^ ± 34.82	74.52	—

*The net AUC was calculated by subtracting the blank AUC from the AUC of each sample, the standards, and the positive control. Final ORAC values were expressed as the equivalent concentration of Trolox (TE) that gives the same level of antioxidant activity as the samples at 20 *μ*g/mL. **Means with different alphabets are statistically significant.

**Table 2:** Compounds tentatively identified in the chloroform fraction of *Centratherum anthelminticum *(CACF).

Peak number	RT^a^	Percentage of the peak^b^	Molecular weight	Molecular formula	Similarity index	Compound^c^
1	5.279	5.97	116	C_6_H_12_O_2_	89	2-Pentanone-4-hydroxy-4-methyl
2	8.086	29.04	172	C_7_H_12_N_2_OS	92	2-Morpholinoethyl isothiocyanate
3	11.278	5.97	180	C_6_H_12_O_6_	85	d-Allose
4	11.547	3.62	346	C_22_H_34_O_3_	65	Drostanolone AC
5	11.650	1.62	162	C_10_H_10_O_2_	62	3(2H)-Benzofuranone, 2,6-dimethyl
6	15.533	6.36	326	C_21_H_42_O_2_	67	Nonadecanoic acid, ethyl ester
7	17.676	16.48	248	C_18_H_32_	78	1,E-11,Z-13-Octadecatriene
8	17.933	1.85	312	C_20_H_40_O_2_	68	Hexadecanoic acid, 1,1-dimethylethyl ester
9	17.950	0.45	145	C_6_H_11_NO_3_	46	Adipic acid monoamide
10	17.967	2.45	282	C_12_H_26_O_5_S	55	d-Mannitol, 1-thiohexyl
11	18.117	0.83	173	C_7_H_11_NO_2_	45	Ethyl 4-isothiocyanatobutyrate
12	21.450	16.15	340	C_22_H_44_O_2_	85	Octadecanoic acid, butyl ester
13	23.350	1.68	172	C_9_H_16_O_3_	50	Arabino-hetitol, 2,3 : 5,6-dianhydro-1,7-dideoxy-2,6-di-e-methyl
14	23.942	2.62	298	C_17_H_14_O_5_	42	4H-1-Benzopyran-4-one,5-hydroxy-7-methoxy-2-(3-methoxyphenyl)

Total		95.09				

^a^RT: retention time (min).

^b^Relative area percentage (peak area relative to the total peak area percentage).

^c^Compounds listed in order of their relative area percentage.
